# Contact Sensitization in Adults With Atopic Dermatitis: A 21‐Year Single‐Center Tertiary Experience

**DOI:** 10.1111/cod.70086

**Published:** 2026-01-29

**Authors:** Francesca Caroppo, Anna Zambello, Giulia Biolo, Fortunato Cassalia, Laura Ventura, Anna Belloni Fortina

**Affiliations:** ^1^ Dermatology Unit, Department of Medicine (DIMED) University of Padua Padua Italy; ^2^ Paediatric Dermatology Regional Center, Department of Women's and Children's Health (SDB) University of Padua Padua Italy; ^3^ Department of Statistics University of Padua Padua Italy

**Keywords:** allergic contact dermatitis, atopic dermatitis, contact sensitisation, formaldehyde, fragrances, isothiazolinones, patch testing, rubber accelerators

## Abstract

**Background:**

Atopic dermatitis is a common chronic inflammatory skin condition. Data on contact sensitization in adults with atopic dermatitis remain limited.

**Objectives:**

To investigate the prevalence of contact sensitization in adults with and without AD:

**Patients/Methods:**

A monocentric, retrospective study analysed 8967 adults (≥ 16 years) patch‐tested at the University of Padua (1997–2018). Patients were tested with a standard baseline series (44 allergens). 540 (6.0%) patients had atopic dermatitis; 8273 (92.3%) did not; 154 (1.7%) had missing/uncertain AD status and were excluded from subgroup comparisons.

**Results:**

Contact sensitization prevalence was 65.4% in both groups. Adults with atopic dermatitis showed significantly higher rates of sensitization to formaldehyde (*p* < 0.00001), HICC (hydroxyisohexyl 3‐cyclohexene carboxaldehyde) (*p* = 0.04), thiuram mix (*p* = 0.03), and carba mix (*p* = 0.004). Non‐AD patients showed significantly higher sensitization to nickel (*p* = 0.001), disperse blue (*p* = 0.006), and primin (*p* = 0.05). Nickel was the most frequent allergen (21.30% AD; 27.59% non‐AD).

**Conclusions:**

Higher prevalence of contact sensitization to specific allergens in adults with atopic dermatitis suggests a tailored patch test series including allergens from rubber, fragrance, and preservative groups. The lower prevalence of metal sensitization in patients with atopic dermatitis may reflect distinct immunological mechanisms that warrant further research.

## Highlights

- Adults with AD show higher sensitization to rubber and preservatives.
- Nickel sensitization is significantly lower in AD than non‐AD adults.
- Suggest tailored patch test series for AD patients.

## Graphical abstract

A graphical abstract has been included as supplemental material.

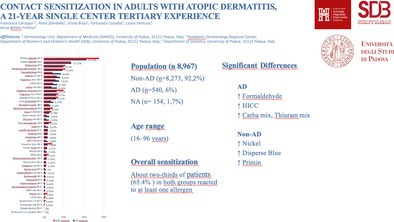

**Abbreviations**
ACD: Allergic contact dermatitis
AD: Atopic dermatitis
APT: atopy patch test
CAPB: cocamidopropyl betaine
ESCD: European Society of Contact Dermatitis
HICC: hydroxyisohexyl 3‐cyclohexene carboxaldehyde (Lyral^®^)
MBT: mercaptobenzothiazole
MDA: 4,4′‐diaminodiphenylmethane
MDGN: methyldibromoglutaronitrile
MI: methylisothiazolinone
PT: Patch test

## 1 Introduction

Allergic contact dermatitis (ACD) is a T cell–mediated type IV hypersensitivity reaction triggered by cutaneous exposure to contact allergens [1].

Atopic dermatitis (AD), also known as atopic eczema, is a common chronic inflammatory dermatosis that usually begins in childhood but may persist into or arise de novo in adulthood.

Although the characteristics of paediatric AD have been extensively investigated, an increasing body of literature highlights the interplay between AD and contact sensitization (CS), with a clinically relevant subset of patients developing ACD.

A recent nationwide, population‐based survey of 40 007 Danish adults reported a lifetime AD prevalence of 9.0%; nearly one‐third of those affected described moderate‐to‐severe disease. AD was independently associated with both hand eczema and positive patch test reactions. The subgroup with both AD and hand eczema showed particularly high rates of contact sensitization and psychiatric comorbidities, highlighting their clinical vulnerability [2].

Given this context, we analysed 21 years of patch‐test data collected at a single tertiary referral centre, comparing adults with and without AD to identify the most frequently implicated allergens and explore potential differences in sensitisation profiles.

## 2 Methods

**FIGURE 1:**
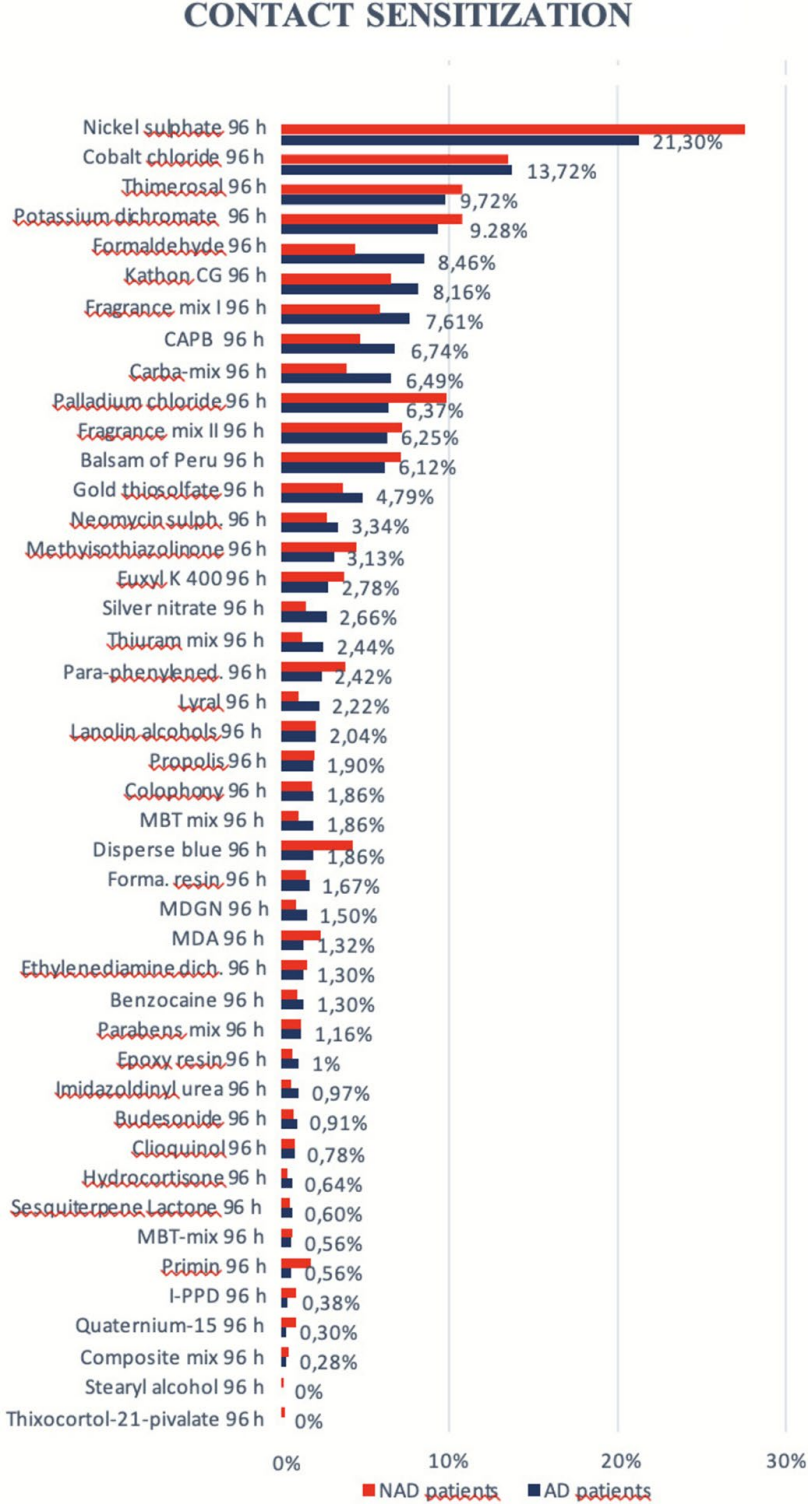
Ranking in decreasing order of contact sensitization at 96 h to different tested allergens. Comparison between AD and NAD patients.

We conducted a retrospective, single‐centre study including all patients aged 16–96 years who underwent patch testing for suspected allergic contact dermatitis (ACD) at the University of Padua between 1997 and 2018. Atopic dermatitis (AD) was diagnosed according to the U.K. Working Party criteria [3], and cases with incomplete documentation were excluded. Overall, 8967 individuals were patch‐tested with a baseline series of 44 allergens: 540 (6.0%) had AD and 8273 (92.3%) did not; an additional 154 (1.7%) had missing/uncertain AD status and were excluded from subgroup analyses (Figure 1).

Patch tests were applied on unaffected dorsal skin and read at 48 and 96 h. The day‐4 reading (96 h) was considered the primary endpoint, in accordance with ESCD guidelines [4]. Testing was deferred if active lesions were present on the back. Patch testing was performed from October to May using Finn Chambers on Scanpor tape, and reactions were scored according to ESCD criteria; only +, ++, or +++ reactions were considered positive, whereas doubtful reactions (?) were classified as negative.

The baseline series was used throughout the study period, although its composition evolved over time according to ESCD/SIDAPA recommendations (e.g., thimerosal removed in 2015; methylisothiazolinone [MI] tested separately from 2018). Absolute numbers of positive reactions are reported in Table 1 and frequencies with *p*‐values in Table 1.

Antihistamines were not considered a contraindication. Topical corticosteroids were withheld from the back for at least 7 days, systemic corticosteroids were discontinued for ≥ 4 weeks, and phototherapy was stopped for ≥ 2 weeks prior to testing. When clinically feasible, systemic immunosuppressants and biologics were discontinued according to standard washout periods.

### 2.1 Statistical Analysis

Data were compiled and merged into a single Excel 2016 database over the years, and statistical analysis was performed using R software (www.r‐project.org). The study was observational, monocentric, and retrospective.

Quantitative variables were expressed as mean (or median) ± standard deviation, and qualitative variables as percentages.

Group comparisons of quantitative variables were performed using Student's *t*‐test, while associations between categorical variables were analysed using Pearson's chi‐square or Fisher's exact test as appropriate.

To evaluate the association between AD and other covariates, a logistic regression model was applied. Statistical significance was defined as *p* < 0.05 for all tests.

## 3 Results

Data from 8967 patients were analysed, including 5949 (66.3%) females and 3018 (33.7%) males. The average age was 43.3 ± 17.0 years. Patients with AD accounted for 540 (6.0%) of the total cohort: of these, 353 (65.5%) were female and 187 (34.5%) male, with an average age of 29.5 ± 12.3 years.

The comparison group without AD (NAD) included 8273 (93.5%) patients: among them, 5504 were female and 2769 were male, with a mean age of 44.2 ± 16.8 years.

An additional 154 patients were classified as “NA” (not applicable) due to missing or uncertain AD data. In our study, 353 AD patients and 5413 NAD patients tested positive for at least one allergen, corresponding to an overall prevalence of 65.4% in both groups (see Table 1). In the entire cohort, the 10 most frequently detected allergens were: nickel (27.23%), cobalt chloride (13.62%), potassium dichromate (10.83%), thimerosal (10.66%), palladium (9.52%), *Myroxylon pereirae* resin (7.10%), fragrance mix II (7.00%), Methylchloroisothiazolinone/Methylisothiazolinone (6.55%), fragrance mix I (5.99%), and cocamidopropyl betaine (4.78%).

Separate analyses of the AD and NAD groups revealed different allergen distribution patterns. In the AD group, the most frequent allergens were: Nickel (21.30%), Cobalt chloride (13.72%), Thimerosal (9.72%), Potassium dichromate (“chrome” replaced for clarity) (9.28%), Formaldehyde (8.46%), Methylchloroisothiazolinone/Methylisothiazolinone (8.16%), Fragrance mix I (7.60%), Cocamidopropylbetaine (6.74%), Carba mix (6.49%), Palladium (6.37%).

In the NAD group, the top allergens were: Nickel (27.59%), Cobalt chloride (13.51%), Thimerosal (10.77%), Potassium dichromate (10.77%), Palladium (9.77%), Fragrance mix II (7.15%), *Myroxylon pereirae* resin (7.10%), Methylchloroisothiazolinone/Methylisothiazolinone (6.51%), Fragrance mix I (5.87%), Cocamidopropylbetaine (4.67%).

The prevalence of CS to each allergen across the AD group, NAD group, and total population is reported in Table 1. Table 1 displays the absolute number of positive patch‐test reactions per allergen and comparative results between groups.

**TABLE 1:** Absolute number (*n*) and frequency (%) of positive patch test reactions at 48 h and 96 h in AD and non‐AD groups, with *p*‐values (allergen concentration and vehicle shown in parentheses).

No	Allergen (concentration; vehicle)	AD 48h *n* (%)	non‐AD 48h *n* (%)	*p* (48h)	AD 96h *n* (%)	non‐AD 96h *n* (%)	*p* (96h)
**1**	THIURAM MIX (1% PET)	11 (2.07)	135 (1.65)	0.4814	13 (2.44)	103 (1.26)	0.02906*
**2**	MYROXYLON PEREIRAE RESIN (BALSAM OF PERU) (25% PET)	27 (5.01)	420 (5.11)	1	33 (6.12)	585 (7.11)	0.4344
**3**	CARBA MIX (3% PET)	12 (5.94)	413 (5.02)	0.3608	35 (6.49)	317 (3.85)	0.004364**
**4**	POTASSIUM DICHROMATE (0.5% PET)	38 (7.06)	587 (7.12)	1	50 (9.28)	888 (10.77)	0.3133
**5**	P‐PHENYLENEDIAMINE (PPD) (1% PET)	12 (1.86)	313 (2.77)	0.6637	13 (2.42)	312 (3.79)	0.1243
**6**	WOOL ALCOHOLS (LANOLIN ALCOHOLS) (30% PET)	13 (1.67)	204 (2.25)	0.4509	11 (2.04)	166 (2.02)	0.875
**7**	ROSIN (COLOPHONY) (20% PET)	8 (1.48)	148 (1.80)	0.736	10 (1.86)	145 (1.77)	0.8652
**8**	NEOMYCIN SULFATE (20% PET)	6 (1.11)	90 (1.10)	0.8324	18 (3.34)	222 (2.70)	0.3414
**9**	N‐ISOPROPYL‐N′‐PHENYL‐P‐PHENYLENEDIAMINE (IPPD) (0.1% PET)	5 (0.93)	71 (0.87)	0.8091	2 (0.38)	70 (0.86)	0.3244
**10**	ETHYLENEDIAMINE DIHYDROCHLORIDE (1% PET)	9 (0.67)	124 (1.51)	0.7151	7 (1.30)	125 (1.52)	0.855
**11**	EPOXY RESIN (BISPHENOL A DIGLYCIDYL ETHER RESIN) (1% PET)	4 (0.73)	50 (0.61)	0.5729	5 (1.00)	53 (0.66)	0.3974
**12**	MERCAPTOBENZOTHIAZOLE (MBT) (2% PET)	7 (1.36)	60 (0.75)	0.1222	10 (1.86)	83 (1.01)	0.07754
**13**	P‐TERT‐BUTYLPHENOL FORMALDEHYDE RESIN (PTBP‐FR) (1% PET)	10 (1.86)	95 (1.16)	0.1495	9 (1.67)	121 (1.47)	0.7109
**14**	COBALT(II) CHLORIDE (1% PET)	51 (9.44)	830 (10.07)	0.7114	74 (13.72)	1114 (13.51)	0.8965
**15**	FRAGRANCE MIX I (FM I) (1% PET)	30 (5.57)	340 (4.13)	0.1202	41 (7.61)	483 (5.87)	0.1099
**16**	BENZOCAINE (5% PET)	12 (2.23)	122 (1.49)	0.1996	7 (1.30)	74 (0.90)	0.3464
**17**	PARABENS MIX (16% PET)	6 (1.11)	117 (1.43)	0.7056	6 (1.16)	95 (1.18)	1
**18**	NICKEL SULFATE (5% PET)	89 (17.10)	1632 (20.13)	0.1124	106 (21.30)	2271 (27.59)	0.001374**
**19**	DISPERSE BLUE 124 (1% PET)	10 (1.86)	255 (3.10)	0.118	10 (1.86)	345 (4.19)	0.006314**
**20**	MERCAPTO MIX (MBT MIX) (PET)	1 (0.28)	52 (0.90)	0.3692	2 (0.56)	36 (0.62)	1
**21**	CLIOQUINOL (QUINOFORM/VIOFORM) (5% PET)	2 (0.66)	38 (0.66)	0.6903	1 (0.78)	42 (0.80)	0.7216
**22**	QUATERNIUM‐15 (1% PET)	3 (0.83)	38 (0.66)	0.7317	1 (0.30)	46 (0.83)	0.5207
**23**	4,4′‐DIAMINODIPHENYLMETHANE (MDA) (0.5% PET)	7 (1.38)	162 (2.01)	0.4097	7 (1.32)	190 (2.32)	0.1721
**24**	METHYLDIBROMO GLUTARONITRILE/PHENOXYETHANOL (MDBGN/PE) (1.5% PET)	14 (2.60)	266 (3.24)	0.526	15 (2.78)	305 (3.71)	0.3413
**25**	THIMEROSAL (0.1% PET)	26 (5.16)	509 (6.34)	0.3428	49 (9.72)	865 (10.77)	0.5041
**26**	FORMALDEHYDE (1% in water)	31 (5.83)	252 (3.07)	0.001363**	45 (8.46)	357 (4.35)	0.00001****
**27**	PRIMIN (0.01% PET)	3 (0.56)	134 (0.63)	0.06774*	3 (0.56)	140 (1.71)	0.04928*
**28**	METHYLCHLOROISOTHIAZOLINONE/METHYLISOTHIAZOLINONE (MCI/MI) (0.02% in water)	34 (6.31)	368 (4.47)	0.05498*	42 (8.16)	521 (6.51)	0.1434
**29**	SESQUITERPENE LACTONE MIX (SLM) (0.1% PET)	1 (0.30)	9 (0.22)	0.5375	2 (0.60)	20 (0.49)	0.6768
**30**	COCAMIDOPROPYL BETAINE (CAPB) (1% in water)	14 (3.26)	173 (3.08)	0.7727	29 (6.74)	263 (4.67)	0.06073
**31**	BUDESONIDE (0.01% PET)	1 (0.30)	8 (0.19)	0.4902	3 (0.91)	28 (0.67)	0.4864
**32**	TIXOCORTOL PIVALATE (0.1% PET)	0 (0.00)	2 (0.05)	1	0 (0.00)	10 (0.24)	1
**33**	HYDROCORTISONE (1% PET)	0 (0.00)	4 (0.10)	1	2 (0.64)	16 (0.38)	0.3499
**34**	STEARYL ALCOHOL (30% PET)	0 (0.00)	1 (0.03)	1	0 (0.00)	6 (0.15)	1
**35**	COMPOSITAE MIX (COMPOSITAE MIX) (5% PET)	2 (0.56)	9 (0.21)	0.192	1 (0.28)	18 (0.41)	1
**36**	IMIDAZOLIDINYL UREA (2% PET)	1 (0.65)	11 (0.24)	0.1957	3 (0.97)	22 (0.53)	0.2417
**37**	PROPOLIS (10% PET)	4 (1.27)	26 (0.63)	0.157	6 (1.90)	81 (1.95)	1
**38**	SILVER NITRATE (1% PET)	0 (0.00)	19 (0.46)	0.3932	9 (2.66)	60 (1.45)	0.1015
**39**	PALLADIUM(II) CHLORIDE (2% PET)	3 (0.96)	115 (2.94)	0.04706*	20 (6.37)	382 (9.77)	0.0566
**40**	GOLD SODIUM THIOSULFATE (0.5% PET)	9 (2.88)	79 (2.02)	0.3008	15 (4.79)	142 (3.63)	0.2775
**41**	HYDROXYISOHEXYL 3‐CYCLOHEXENE CARBOXALDEHYDE (HICC) (FORMERLY LYRAL) (5% PET)	6 (1.90)	19 (0.55)	0.01429*	7 (2.22)	33 (0.95)	0.04381*
**42**	METHYLDIBROMO GLUTARONITRILE (MDBGN) (0.5% PET)	4 (1.20)	15 (0.44)	0.06688	5 (1.50)	28 (0.81)	0.1903
**43**	FRAGRANCE MIX II (FM II) (14% PET)	1 (1.56)	15 (3.35)	0.7062	4 (6.25)	32 (7.15)	1
**44**	METHYLISOTHIAZOLINONE (MI)	0 (0.00)	8 (1.79)	0.6042	2 (3.13)	20 (4.47)	1

## 4 Discussion

AD skin is characterised by filaggrin deficiency, reduced ceramide levels, and tight junction disruption, all of which facilitate hapten penetration. This impaired barrier, along with chronic scratching, creates a pro‐sensitising microenvironment.

Immunologically, acute AD lesions are dominated by a Th2 cytokine profile (IL‐4, IL‐13), whereas chronic AD and ACD show Th1/Th17 skewing (IFN‐γ, IL‐17, IL‐22). This cytokine switch may explain why only a subset of atopic patients becomes contact‐sensitised.

For clarity, “contact sensitisation” (CS) refers to a positive patch‐test reaction, whereas “allergic contact dermatitis” (ACD) refers to clinically relevant eczema caused by allergen exposure.

Identifying concomitant ACD is essential, as avoidance of a single allergen can lead to significant improvement in so‐called “refractory AD.”

In clinical practice, patch testing should be considered in adults with atypical distribution, therapy‐resistant eczema, or occupational exposure, even when eczema is labelled “atopic.”

Although the relationship between AD and ACD remains controversial, several studies suggest that patients with AD may be at increased risk of developing ACD [5, 6, 7]. However, as noted by Owen et al., ACD develops only in a subset of AD patients, and routine patch testing is not universally recommended for all individuals with AD [8].

### 4.1 Metals

Nickel was the most frequent allergen in both groups, but its prevalence was significantly lower in AD than in NAD patients. This finding aligns with reports suggesting a reduced risk of nickel sensitization in AD due to IL‐4/IL‐13–mediated TLR‐4 downregulation and reduced IL‐17 activity [9].

Teo et al. hypothesized that this may be due to immunological mechanisms: nickel can directly activate TLR‐4, which is downregulated by AD‐associated cytokines IL‐4 and IL‐13 [10].

Moreover, IL‐17, a proinflammatory cytokine involved in the development of allergic responses, has been shown to play a critical role in nickel‐induced sensitization. Reduced IL‐17 production in the Th2‐skewed environment typical of AD may further contribute to decreased sensitization to nickel [11].

In addition to immunological factors, behavioural and regulatory influences may also explain the difference. Patients with AD may avoid nickel exposure due to public health campaigns discouraging piercings and promoting the use of “nickel‐free” products.

The European Nickel Directive (1994/27/EC), in force since 2001, limits nickel release from metal objects. EU restrictions on nickel release from items intended for direct and prolonged skin contact may have reduced population exposure over time.

Another explanation may be that patch testing is sometimes performed in AD patients as a diagnostic exclusion tool in cases of atypical or therapy‐refractory eczema, even in the absence of clear clinical suspicion. In contrast, NAD patients are often tested when ACD is strongly suspected, potentially increasing their rate of positive results [7, 8].

A similar trend was observed for palladium (*p* = 0.05).

In contrast, there were no significant differences for CS to cobalt chloride or potassium dichromate.

Interestingly, higher—but not statistically significant—prevalence was found in AD patients for gold thiosulfate and silver nitrate.

The industrial background of North‐East Italy, with high exposure to metal‐releasing costume jewellery and accessories, may contribute to the high CS rates in the general population. AD patients, conversely, may adopt avoidance behaviours from an early age, particularly regarding nickel and textile‐related allergens, which could explain their lower sensitisation rates.

### 4.2 Preservatives

Thimerosal (also known as merthiolate or mercurochrome) was the fourth most common allergen in both groups, with no significant difference. Thimerosal is a mercury‐derived preservative formerly used in vaccines and topical preparations. Since 2000, its use has decreased markedly in both the US and EU due to safety concerns [9].

Although still found in some ophthalmic and contact lens solutions, it has been removed from standard series in many countries, including our center [9, 10]. Despite high patch‐test positivity, its clinical relevance is limited, often reflecting past sensitisation or false positives, especially in the absence of active exposure [11].

Formaldehyde showed a significantly higher prevalence of contact sensitization (CS) in the AD group compared to the NAD group.

Although we used a 1% concentration in patch testing, recent European guidelines recommend 2% to reduce false negatives [12].

Formaldehyde‐releasing preservatives remain widespread in cosmetics and household products, despite formaldehyde itself being less frequently used. Their patch test positivity was low, yet clinical relevance is likely underestimated, as nearly half of formaldehyde‐releasing cosmetics do not list it on the INCI label [13].

Even low concentrations can exacerbate dermatitis, especially in patients with AD. Reported CS prevalence to formaldehyde ranges from 2% to 9% across Europe, with 3.3% observed in northeastern Italy [14]. Labeling regulations require declaration only above 500 ppm (0.05%), leaving smaller amounts undisclosed [15].

In our series, the prevalence of CS to:

- Quaternium‐15 was 0.78%
- Imidazolidinyl urea was 0.56%. No significant differences were found between AD and NAD groups (*p* > 0.05), in line with European data [16].

Methylchloroisothiazolinone/Methylisothiazolinone sensitization was more frequent in the AD group, although the difference was not statistically significant (*p* > 0.05).

Methylchloroisothiazolinone/Methylisothiazolinone are widely used preservatives found in rinse‐off and leave‐on cosmetics, household cleaning agents, paints, adhesives, and industrial products. Their widespread use has led to a global increase in contact sensitization, particularly between 2005 and 2015, when sensitization rates rose sharply before regulatory measures were implemented [17, 18].

Methylisothiazolinone has been identified as one of the most common emerging contact allergens in Europe and North America, with particularly high sensitization rates in individuals with atopic dermatitis, likely due to increased product use and an impaired skin barrier [19, 20]. In 2017, the European Scientific Committee on Consumer Safety (SCCS) limited Methylisothiazolinone use in leave‐on cosmetics due to the high number of allergic reactions, a move that led to a subsequent reduction in sensitization rates [21].

Despite these regulatory actions, Methylchloroisothiazolinone/Methylisothiazolinone remains relevant allergens because they are still present in many rinse‐off products and industrial materials, with high risk for patients with AD [22].

Paraben mix showed a relatively low prevalence of sensitization in our study. They are considered weak allergens and are rarely associated with clinically significant allergic contact dermatitis (ACD) [23, 24].

Due to their low allergenic potential and long‐standing use, parabens have often been considered safer alternatives to other preservatives. Several large‐scale studies, including the North American Contact Dermatitis Group (NACDG) data, have reported sensitization rates generally below 1%–2% [25, 26]. Nevertheless, they may still cause allergic reactions in sensitised individuals, particularly when applied to broken or eczematous skin.

### 4.3 Rubber

In the rubber group, the most frequent allergen was carba mix, showing with thiuram mix a statistically significant difference between the AD and NAD groups.

Plausible contributing factors include:

(i) prolonged occlusion from gloves or footwear, which may enhance percutaneous absorption of rubber additives through the already compromised atopic skin barrier;

(ii) repeated application of emollients under wraps or bandages, which could dissolve and transfer thiuram/carba accelerators into the skin;

(iii) the Th2‐skewed cytokine milieu characteristic of AD, which may alter cutaneous redox balance, thereby facilitating hapten binding to epidermal proteins.

Although these mechanisms remain hypothetical and the literature is limited in the adult atopic population, collectively they may offer a biological explanation for the increased rubber sensitization observed in our AD cohort.

Most studies examining the relationship between ACD and AD have been conducted in paediatric populations and have identified common allergens including nickel sulfate, cobalt chloride, potassium dichromate, lanolin, neomycin, formaldehyde, sesquiterpene lactones, compositae mixes, and fragrances [19, 21].

A possible reason why no data was reported about these rubber accelerators in children is probably due to the fact that carba mix and thiuram mix have higher reactivity rates with increasing age [22, 23].

The higher incidence in older individuals may be related to longer cumulative exposure to sources such as shoes and gloves, or to relatively greater exposure to rubber components in medical devices (e.g., support stockings).

### 4.4 Fragrances

Among fragrance allergens, no statistically significant differences were observed between the AD and NAD groups: the high prevalence of fragrance sensitization in both populations highlights the clinical relevance of this allergen class, especially in the context of atopic skin.

Fragrance mix I (containing compounds such as cinnamal, eugenol, isoeugenol, and hydroxycitronellal) and fragrance mix II (containing hydroxyisohexyl 3‐cyclohexene carboxaldehyde [Lyral], citral, and farnesol, among others) include substances frequently used in cosmetics, personal care products, topical medications, and household detergents [27, 28].

*Myroxylon pereirae* resin, commonly found in perfumes and scented products, is a complex natural extract containing multiple sensitising compounds such as cinnamic acid derivatives and benzyl benzoate. Although often considered a marker for fragrance allergy, it can also reflect sensitization to structurally unrelated allergens [29].

Patients with AD may be particularly vulnerable to fragrance allergens due to both intrinsic immune dysregulation and environmental exposure. Notably, many products marketed as “hypoallergenic” or “for sensitive skin” may still contain fragrance compounds or their derivatives, increasing the risk of chronic exposure and sensitisation [30, 31].

Furthermore, some fragrance allergens are not required to be listed individually on ingredient labels unless present above a certain threshold (0.001% in leave‐on products and 0.01% in rinse‐off products), making avoidance difficult for sensitised individuals. Hidden or undeclared fragrance allergens, particularly in emollients and baby care products, further complicate management [32].

Several studies have confirmed that fragrance mix I is among the top allergens in AD populations and that early sensitisation may predispose patients to lifelong fragrance intolerance [33, 34, 35].

Given the frequent use of fragranced products and the challenges in avoidance, fragrance allergy represents a significant problem in the atopic population and should be thoroughly investigated in all AD patients with chronic or treatment‐resistant eczema, particularly involving the face, neck, or flexural areas, classic sites of fragrance‐induced dermatitis [34].

### 4.5 Cocamidopropylbetaine

We observed different rates of CS to CAPB between the AD and NAD groups, though the difference did not reach statistical significance.

Surfactants are known to impair skin barrier function and exacerbate inflammation in patients with ACD and AD [36]. To assess differential reactivity to surfactants, Shaughnessy et al. (2014) analysed 1674 patients with and without AD, showing an association between CS to CAPB and a history of AD and concluded that children with AD should not be exposed to this allergen [37].

These findings were supported by further studies in paediatric populations [38]. Collis et al. reported that all reactions to CAPB occurred exclusively in patients with AD [39].

CAPB is widely used in shampoos, detergents, contact lens solutions, and antiseptics and is often found in products labelled “hypoallergenic,” many of which are marketed specifically for sensitive skin and for children with ACD and AD [40]. Given the higher likelihood of CAPB sensitization in AD patients, this allergen should be avoided from childhood through adulthood.

### 4.6 Topical Drugs

No statistically significant differences were found in CS to topical medications between AD and NAD groups. However, higher prevalences were observed in the AD group for neomycin, benzocaine, budesonide, and hydrocortisone.

Thixocortol had a low overall prevalence of 0.2%, with no positive cases in the AD group and only 10 cases in the NAD group.

### 4.7 Disperse Blue

This allergen refers specifically to Disperse Blue 124, a dye used in nylon, shirts, and synthetic fabrics. On fabric fibres, particularly synthetic ones, the dye is loosely bound and can be released onto the skin.

Patients sensitised to this allergen are advised to avoid dark‐coloured and synthetic clothing.

In our study, the prevalence of CS to Disperse Blue was significantly higher in the NAD group compared to the AD group (*p* = 0.006).

This may be because patients with AD are often advised to wear light cotton or natural fibre clothing and to avoid dark, synthetic garments.

Such preventive recommendations, particularly those concerning nickel (used in fasteners in dark clothes) and textile dyes, may be effective in reducing sensitization in adult AD patients.

### 4.8 Primin

In our sample, the overall prevalence of CS to primin in the NAD group was higher than in the AD group.

Primin is a potent contact allergen present in the ornamental plant *Primula obconica Hance*, and it was included in the European Standard Series (ESS) in 1984 [41].

In 2000, a “primin‐free” variant of *P. obconica* was introduced to reduce the risk of contact dermatitis. Since then, cultivation of primin‐free *P. obconica* has increased significantly. Several studies have shown a decline in ACD related to this plant [42]. A Northern Italian study also found decreasing rates of primin sensitization, with persistence mainly among retirees and domestic workers [43].

The lower prevalence of CS to primin in the AD group may be attributable to the age difference between the groups.

It is hypothesized that in patients with severe AD, there may be a threshold beyond which chronic barrier impairment leads to immune tolerance rather than sensitization.

An additional explanation could be that AD patients with coexisting respiratory allergies to pollen may avoid plant contact, thereby reducing exposure [8].

### 4.9 Compositae Mix

In contrast to findings in the paediatric population from the Paduan case series, we did not observe a significant difference in CS to compositae mix in adults with AD.

There are several potential pitfalls to consider when performing patch testing in patients with atopic dermatitis.

AD patients have a lower threshold for irritation, which may lead to a higher rate of irritant or false‐positive reactions to common contact allergens, particularly metals, fragrances, formaldehyde, and lanolin [43].

In addition, clinically relevant positive reactions in AD patients may appear weaker and can be misinterpreted as irritant reactions, potentially leading to false‐negative conclusions.

This is partly due to the fact that AD patients often do not exhibit the typical “crescendo” pattern of increasing reactivity between patch test readings (e.g., from + at 48 h to +++ at 96 h), which is commonly seen in classic positive reactions.

Moreover, positive reactions may be inherently weaker in individuals with AD, especially in those with more severe disease.

Paradoxically, active AD can also lead to false‐negative patch test results, even when tests are applied to clinically unaffected skin and in the absence of systemic immunosuppressive therapy [44].

This suggests that patch testing in AD patients—particularly those with severe disease—requires careful interpretation, ideally by experienced clinicians familiar with the nuances of testing in this population.

## 5 Limitations

Despite its strengths, this study has several limitations that should be acknowledged to improve its scientific validity.

First, the retrospective design may introduce selection bias and limits the ability to establish causal relationships. As a single‐centre study, the findings may reflect local patterns of allergen exposure, which could reduce their generalisability to broader populations.

Moreover, atopic dermatitis (AD) diagnoses were based on medical records rather than consistent, standardised diagnostic criteria, potentially affecting the accuracy of patient classification. The study also lacked detailed information on occupational and environmental exposures—key factors in assessing the risk of contact sensitization.

Future prospective, multicentre studies using standardised diagnostic criteria and comprehensive exposure assessments are needed to validate and expand upon these findings.

## 6 Conclusions

In our study, statistically significant differences in contact sensitization (CS) to several allergens were observed between the AD and NAD groups. Formaldehyde, HICC (Lyral®), thiuram mix, and carba mix were significantly more frequent in the AD group.

These findings may contribute to the development of a patch‐test screening series tailored for patients with atopic dermatitis. In our geographical area, such a series should include allergens from the rubber category—particularly carba mix and thiuram mix—as well as fragrance mix I and II, HICC, preservatives, and cocamidopropyl betaine (CAPB).

A lower prevalence of CS to nickel sulphate and palladium was found in the AD group compared to the NAD group. This difference may reflect underlying immunological mechanisms specific to AD and warrants further investigation.

Patch‐test screening series are not universally standardised and often vary by region according to local patterns of allergen exposure. Currently, no specific series exists for AD patients, and there is a lack of evidence‐based guidelines on recommended allergens in this population.

The differences in CS prevalence observed in our study likely reflect multiple factors, particularly regional exposure trends and the frequency and duration of allergen contact.

Our data may help inform the development of a standard patch‐test series dedicated to adult patients with AD or a personal history of atopy. Compared to the 2016 SIDAPA baseline series—currently the most widely used in Italy—we recommend including at least two additional allergens: carba mix and CAPB, which were both relevant in our cohort.

Finally, the selection of allergens should always be individualised, based on factors such as geographic region, occupation, hobbies, and personal care habits. We recommend that patch‐test screening in AD patients should include allergens from the rubber category (carba and thiuram mix), fragrances (FM I, FM II, HICC), preservatives, and cocamidopropyl betaine.

## Author Contributions

**Francesca Caroppo:** conceptualization, data curation and writing – original draft. **Anna Zambello, Giulia Biolo and Fortunato Cassalia:** data curation, methodology. **Laura Ventura:** statistical analysis and data interpretation. **Anna Belloni Fortina:** conceptualization, supervision, writing – review and editing.

## Funding

The authors have nothing to report.

## Ethics Statement

This study was performed following the Declaration of Helsinki. All participants provided informed consent for publication of anonymized clinical details.

## Conflicts of Interest

The authors declare no conflicts of interest.

## Data Availability

The data that support the findings of this study are available from the corresponding author upon reasonable request.
